# Differentiation of pulmonary complications with extensive ground-glass attenuation on high-resolution CT in immunocompromised patients

**DOI:** 10.1007/s11604-021-01122-8

**Published:** 2021-05-04

**Authors:** Yoshie Kunihiro, Nobuyuki Tanaka, Reo Kawano, Tsuneo Matsumoto, Taiga Kobayashi, Toshiaki Yujiri, Makoto Kubo, Toshikazu Gondo, Katsuyoshi Ito

**Affiliations:** 1 Present Address: Department of Radiology, Yamaguchi University Graduate School of Medicine, 1-1-1 Minamikogushi, Ube, Yamaguchi 755-8505 Japan; 2 Department of Radiology, National Hospital Organization Yamaguchi - Ube Medical Center, 685 Higashikiwa, Ube, Yamaguchi 755-0241 Japan; 3 Center for Integrated Medical Research, Hiroshima University Hospital, Kasumi 1-2-3 Minami-ku, Hiroshima, 734-8551 Japan; 4 Division of Endocrinology, Metabolism, Hematological Science and Therapeutics, Yamaguchi University Graduate School of Medicine, 1-1-1 Minamikogushi, Ube, Yamaguchi 755-8505 Japan; 5 Department of Medicine and Clinical Science, Yamaguchi University Graduate School of Medicine, 1-1-1 Minamikogushi, Ube, Yamaguchi 755-8505 Japan; 6 Division of Pathology, Fujisawa City Hospital, 2-6-1 Fujisawa, Fujisawa, Kanagawa 251-8550 Japan

**Keywords:** X-ray computed tomography, Ground-glass opacity, Immunocompromised host, Multivariate analysis

## Abstract

**Purpose:**

The purpose of this study was to compare the high-resolution CT (HRCT) findings of pulmonary infectious and noninfectious complications with extensive ground-glass attenuation (GGA) in immunocompromised patients.

**Materials and methods:**

One hundred fifty-two immunocompromised patients with pulmonary complications that showed extensive GGA (> 50% of the whole lung on HRCT) were included in this study. The diagnoses of the 152 patients were as follows: pneumocystis pneumonia (PCP), *n* = 82; drug-induced pneumonia, *n* = 38; bacterial pneumonia, *n* = 9; cytomegalovirus pneumonia, *n* = 6; idiopathic pneumonia syndrome, *n* = 6; diffuse alveolar hemorrhage (DAH), *n* = 4; fungal infection, *n *= 3; tuberculosis, *n* = 2 and pulmonary edema, *n* = 2. Two chest radiologists retrospectively evaluated the CT criteria, which consisted of 12 findings.

**Results:**

The nodule (*p* = 0.015), the bronchovascular bundle (BVB) thickening (*p* = 0.001), and the interlobular septum (ILS) thickening (*p* = 0.002) were significantly infrequent in PCP. The ILS thickening was significantly frequent in drug-induced pneumonia (*p* < 0.001) though it was also frequent in other noninfectious and infectious diseases. The BVB thickening was significantly frequent in bacterial pneumonia (*p* = 0.005). The nodule was significantly frequent in DAH (*p* = 0.049).

**Conclusion:**

Nodules, BVB thickening, and ILS thickening could be useful HRCT findings for the differential diagnosis of pulmonary complications in immunocompromised patients with extensive GGA.

## Introduction

Ground-glass attenuation (GGA) or ground-glass opacity (GGO) is defined as increased attenuation of the lung parenchyma without obscuration of the pulmonary vessels on high-resolution CT (HRCT) and is caused by a wide variety of interstitial and alveolar diseases [1, 2]. In immunocompromised patients, pulmonary complications, especially those which show extensive GGA on HRCT, could be major causes of morbidity and mortality in immunocompromised patients. Acute or subacute diffuse GGA may be caused by opportunistic infections, such as pneumocystis pneumonia (PCP), cytomegalovirus pneumonia (CMV-P), and noninfectious diseases, including drug-induced pneumonia, pulmonary edema, diffuse alveolar hemorrhage (DAH), and acute respiratory distress syndrome (ARDS) [3]. The underlying causes of ARDS could include sepsis, pneumonia, and other infectious or noninfectious conditions. ARDS shows diffuse alveolar damage (DAD) pattern on histology. In patients treated with hematopoietic stem cell transplantation (HSCT), idiopathic pneumonia syndrome (IPS) could also occur as diffuse lung injury [4, 5]. It is important to distinguish between these diseases because the therapeutic strategies are different; however, it is challenging due to the similarities of the clinical and HRCT findings.

The objective of this study was to evaluate and compare the HRCT findings of pulmonary complications with extensive GGA on HRCT in immunocompromised patients and identify indicators that can be used for differentiation between these complications.

## Materials and methods

This study was approved by the institutional review board of our hospital, and the requirement for written informed consent was waived because of the retrospective design.

### Patients

We retrospectively reviewed the CT images of immunocompromised patients with acute chest complications at our hospital from January 1990 to December 2015. Immunocompromised state was defined as having diseases or conditions which cause immunosuppression, or taking immunosuppressive treatment. The selection criteria for this study were as follows: (1) the patients underwent HRCT scans, (2) HRCT showed extensive GGA (> 50% of the whole lung), and (3) only one final diagnosis (with the exception of tumor infiltration) was made for each patient. At first, we identified a total of 1073 patients. Among them, 478 patients were excluded because the lung diseases were not specified in spite of the detailed evaluation of medical records and laboratory findings, including sputum culture, serological tests for several organisms, or the results of bronchoalveolar lavage (BAL), transbronchial lung biopsy (TBLB), blood culture, urinary antigen and autopsy. ARDS cases were also excluded because the causes could be overlapping and they were difficult to specify. Among infectious diseases, 25 patients were excluded because of the existence of co-infections. Then, 83 patients with pulmonary infiltration of tumor (underlying disease) were excluded because it could be difficult to specify the cause of diffuse GGA and exclude other noninfectious or infectious complications in cases who already had pulmonary involvement by malignancy. In addition, 11 patients in whom complications occurred within four weeks after a previous episode were excluded from this study because the previous episode might have influenced the HRCT findings of the current disease. Finally, 324 patients without extensive GGA were excluded. One hundred fifty-two patients (83 males and 69 females; mean age (± standard deviation [SD]): 59.6 ± 16.1 years; range: 3–90 years) were included in this study. Among these, 4 patients had 2 episodes. The final diagnosis of the 152 patients were as follows: PCP (*n* = 82), drug-induced pneumonia (*n* = 38), bacterial pneumonia (*n* = 9), CMV-P (*n* = 6), IPS (*n* = 6), DAH (*n* = 4), fungal infection (*n* = 3), TB (*n* = 2), and pulmonary edema (*n* = 2). Table 1 shows the underlying diseases and the diagnostic methods of all patients (Table 1). Some cases were diagnosed based on the clinical course and these diagnoses were basically determined by physicians according to the detailed analysis of all physical and laboratory findings and responsiveness to therapy. The criteria for the diagnoses according to the clinical course were as follows.

**Table 1:** The patient characteristics and diagnostic methods

	PCP (*n* = 82)	Drug induced pneumonia (*n* = 38)	Bacterial pneumonia (*n* = 9)	CMV-P (*n* = 6)	IPS (*n* = 6)	DAH (*n* = 4)	Fungal infection (*n* = 3)	TB (*n* = 2)	Pulmonary edema (*n* = 2)	*p* value
Age (SD)	59.1 (15.7)	65.4 (12.9)	59.4 (22.5)	49.8 (25.1)	51.8 (13.3)	59.5 (10.6)	72.3 (4.0)	52.5 (17.7)	50.0 (16.3)	0.009
Males, *n* (%)	40 (48.8)	23 (60.5)	8 (88.9)	3 (50.0)	4 (66.7)	2 (50.0)	1 (0.3)	1 (50.0)	1 (50.0)	0.542
Underlying disease or condition, *n* (%)										< 0.001
Hematologic malignancy without HSCT	24 (29.3)	10 (26.3)	2 (22.2)	2 (33.3)	0 (0)	2 (50.0)	1 (33.3)	0 (0)	1 (50.0)	
Hematologic malignancy with HSCT	7 (8.5)	0 (0)	1 (11.1)	2 (33.3)	6 (100)	1 (25.0)	0 (0)	0 (0)	1 (50.0)	
Steroid or other immunosuppressant	46 (56.1)	7 (18.4)	2 (22.2)	2 (33.3)	0 (0)	0 (0)	2 (66.7)	2 (100)	0 (0)	
Solid cancer with chemotherapy	2 (2.4)	21 (55.3)	3 (33.3)	0 (0)	0 (0)	1 (25.0)	0 (0)	0 (0)	0 (0)	
AIDS	3 (3.7)	0 (0)	0 (0)	0 (0)	0 (0)	0 (0)	0 (0)	0 (0)	0 (0)	
Severe burn	0 (0)	0 (0)	1 (11.1)	0 (0)	0 (0)	0 (0)	0 (1.3)	0 ()	0 (0)	
Diagnostic methods, *n* (%)										< 0.001
Sputum culture	9 (11.0)	0 (0)	5 (55.6)	0 (0)	0 (0)	0 (0)	1 (33.3)	1 (50.0)	0 (0)	
Blood culture	0 (0)	0 (0)	3 (33.3)	0 (0)	0 (0)	0 (0)	1 (33.3)	0 (0)	0 (0)	
TBLB or BAL	26 (31.7)	4 (10.5)	1 (11.1)	4 (66.7)	3 (50.0)	2 (50.0)	1 (33.3)	1 (50.0)	0 (0)	
Autopsy	1 (1.2)	0 (0)	0 (0)	2 (33.3)	0 (0)	0 (0)	0 (0)	0 (0)	0 (0)	
Serum marker	46 (56.1)	0 (0)	0 (0)	0 (0)	0 (0)	0 (0)	0 (0)	0 (0)	0 (0)	
DLST	0 (0)	8 (21.1)	0 (0)	0 (0)	0 (0)	0 (0)	0 (0)	0 (0)	0 (0)	
Other clinical course	0 (0)	26 (68.4)	0 (0)	0 (0)	3 (50.0)	2 (50.0)	0 (0)	0 (0)	2 (100)	

*PCP* pneumocystis pneumonia, *CMV-P* cytomegalovirus pneumonia, *IPS* idiopathic pneumonia syndrome, *DAH* diffuse alveolar hemorrhage, *TB* tuberculosis, *HSCT* hematopoietic stem cell transplantation, *AIDS* acquired immunodeficiency syndrome, *TBLB* transbronchial lung biopsy, *BAL* bronchoalveolar lavage, *DLST* drug lymphocyte stimulation test

Serum marker, β-D-glucan > 31 pg/mL in PCP [28]

Drug-induced pneumonia: (1) onset of lung diseases after chemotherapy; (2) immediate responsiveness to newly administrated steroids; and (3) exclusion of infection from the results of sputum culture, and serum antigens or antibodies for specific microorganisms or BAL. Drug-induced pneumonia could progress despite steroid administration especially with DAD pattern; however, the patients with progression were not included in this study because it could be difficult to specify the cause of diffuse GGA and exclude other noninfectious or infectious complications.

DAH: (1) clinical symptoms (massive hemoptysis) under the state of thrombocytopenia.

Pulmonary edema: (1) the presence of heart failure confirmed by echocardiography; and (2) an immediate response to diuretics and/or cardiotonic agents.

IPS: (1) clinically defined by widespread lung injury after HSCT; (2) the absence of infection; and (3) the absence of cardiac, renal or iatrogenic etiology [5].

The subjects recruited for this study were previously reported in other studies [6, 7]. One study included 345 subjects and compared HRCT findings among infectious diseases [6], and the other study included 555 subjects and compared HRCT findings between infectious and non-infectious diseases [7]. The goal of the present study was to compare the findings of patients with extensive GGA on HRCT; thus, the purpose of the current study differs from that of the previous reports [6, 7].

### CT examinations

CT scans were obtained using a TCT-900S (Canon Medical Systems Corporations), a SOMATOM PLUS 4, a Volume Zoom, Somatom Definition, or Somatom Sensation 64 (Siemens). CT scans were obtained at suspended end-inspiratory effort in the supine position, basically without intravenous contrast material. With a TCT-900S scanner, after 10-mm collimation scans were obtained in contiguous 10-mm intervals through the entire chest, all patients underwent HRCT through the region showing abnormal parenchymal findings at 2 mm collimation. With other multislice CT scanners, after contiguous 5-, 7- or 10-mm section CT was performed through the chest, additional HRCT images, consisting of 1- or 2-mm collimated images were obtained at 1-, 2-, 5-, or 10-mm intervals through the abnormal lung parenchyma. In all patients, the scanning parameters were 120–140 kVp and 160–250 effective mAs.

While image data were viewed on hard copy films when CT was performed with a TCT-900S, all image data were interfaced directly to our picture archiving and communication system (PACS) (ShadeQuest; Yokogawa Medical Solutions Corp.), which displayed all image data on monitors (three monitors, 1280 × 1080 matrix, 8-bit viewable gray-scale) when CT was performed using other multislice CT scanners. The monitors were used to view both lung (window width, 1500–1750 HU; window level, − 600 to − 700 HU) and mediastinal (window width, 250–400 HU; window level, 40–50 HU) window images.

### Evaluation of CT images

The CT images were assessed independently by two board-certified chest radiologists (15 and 28 years of experience, respectively) who had no knowledge of the patients’ clinical information other than the fact that all patients were immunocompromised hosts. Discordant results between the two radiologists were resolved by consensus of the same two radiologists.

The presence or absence of the following HRCT findings were coded: (a) crazy-paving pattern, (b) mosaic pattern (mosaic perfusion), (c) airspace consolidation, (d) nodules (e) bronchovascular bundle (BVB) thickening, (f) interlobular septal (ILS) thickening, (g) hilar or mediastinal lymph node (LN) swelling, and (h) pleural effusion.

The presence of GGA was defined if there was a hazy increase in lung opacity without obscuration of vessels. The presence of crazy-paving pattern was recorded if there was superimposition of interlobular or intralobular interstitial thickening within the GGA. Mosaic pattern was defined as sharply demarcated lung areas of inhomogeneous attenuation in which intervening areas of the normal lung were observed between GGAs.

The presence of airspace consolidation was defined if there was increased lung attenuation with obscuration of vessels.

Nodules were classified in size as micro (< 3 mm), small (> 3 to < 10 mm), or large (≥ 10 mm), and in distribution as centrilobular, perilymphatic or random. Centrilobular distribution was defined by the presence of nodules around the peripheral pulmonary bronchial branches or 3–5 mm from the pleura, interlobular septa, pulmonary veins, or relatively proximal pulmonary arteries. Perilymphatic distribution was defined by the presence of nodules around the peribronchovascular interstitium, interlobular septa, and subpleural regions. Random distribution was defined when nodules did not show any specific distribution within the secondary pulmonary lobules.

The overall lesional distribution was classified axially; overall axial distribution was defined as central, peripheral, diffuse or indeterminate and craniocaudal; overall craniocaudal distribution was defined as upper, lower, diffuse or indeterminate.

### Statistical analysis

Interobserver agreement between the 2 radiologists was assessed by calculating the kappa value (*κ* value) for the above-mentioned HRCT findings from (a) to (j): poor (*κ* = 0.00–0.20), fair (*κ* = 0.21–0.40), moderate (*κ* = 0.41–0.60), good (*κ* = 0.61–0.80), or excellent (*κ* = 0.81–1.00) [8].

Each CT finding and CT pattern was compared between the diseases using a chi-squared (*χ*^*2*^) test for independence. Age was compared using the Kruskal Wallis test.

A multiple logistic regression analysis was performed in order to identify significant indicators for differentiation among diseases, for example, between PCP and non-PCP (a combination of drug-induced pneumonia, bacterial pneumonia, CMV-P, IPS, DAH, fungal infection, TB, and pulmonary edema), and between drug-induced pneumonia and non-drug induced pneumonia (a combination of PCP, bacterial pneumonia, CMV-P, IPS, DAH, fungal infection, TB, and pulmonary edema). The forward selection method was used for the multiple logistic regression analysis, and independent variables included all HRCT findings (12 criteria). The area under the curve (AUC) of each model was calculated. All statistical analyses were performed using a commercially available software program (SPSS, version 22.0, IBM). *P* values of < 0.05 were considered to indicate statistical significance.

## Results

**Fig. 1:**
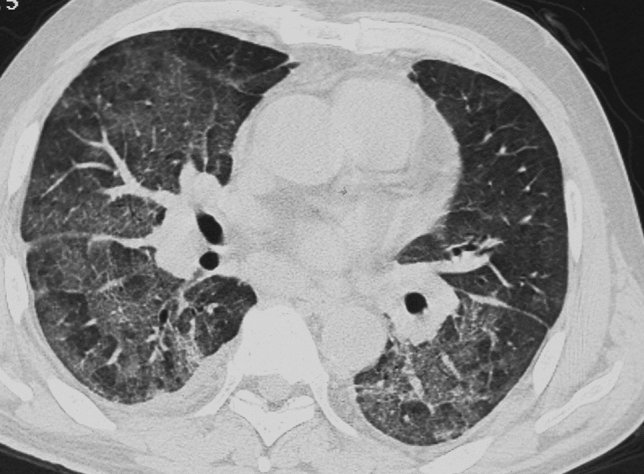
A 60-year-old man with pneumocystis pneumonia under treatment for acute lymphoid leukemia. High-resolution computed tomography shows extensive ground-glass attenuation with a mosaic pattern

**Fig. 2:**
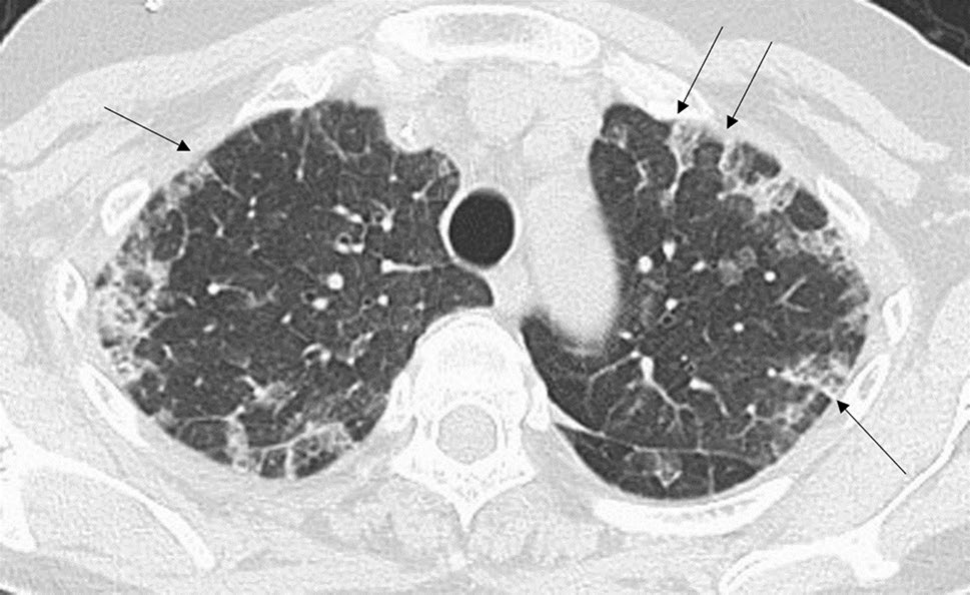
A 61-year-old woman with drug-induced pneumonia under chemotherapy for colon cancer. High-resolution computed tomography shows ground-glass attenuation with outer distribution and thickening of the interlobular septum (arrows)

**Fig. 3:**
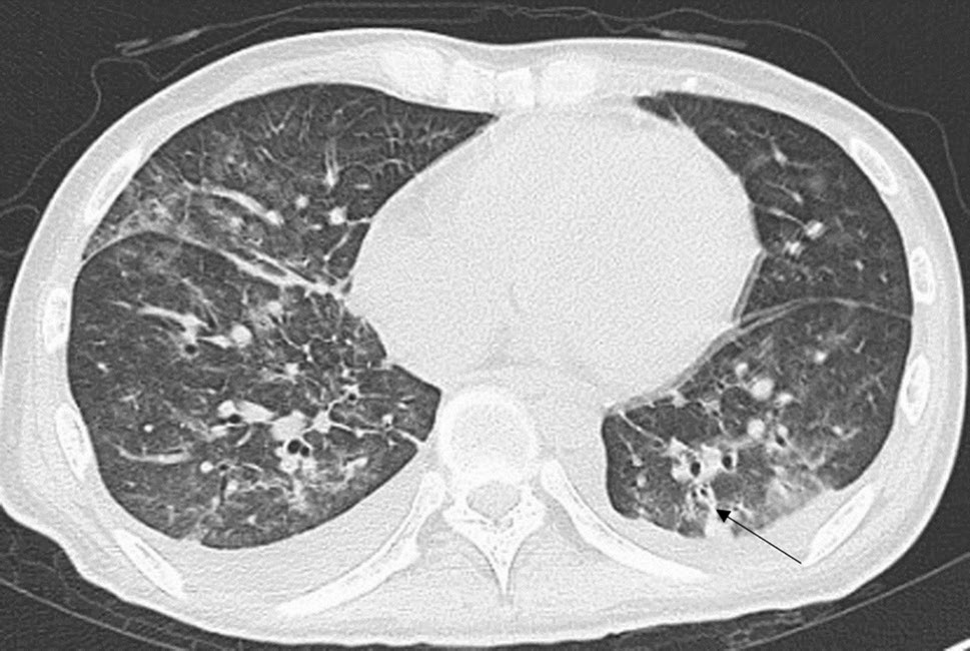
A 29-year-old man with bacterial pneumonia under treatment for acute leukemia. High-resolution computed tomography shows patchy ground-glass attenuation with bronchovascular bundle thickening (arrow)

**Fig. 4:**
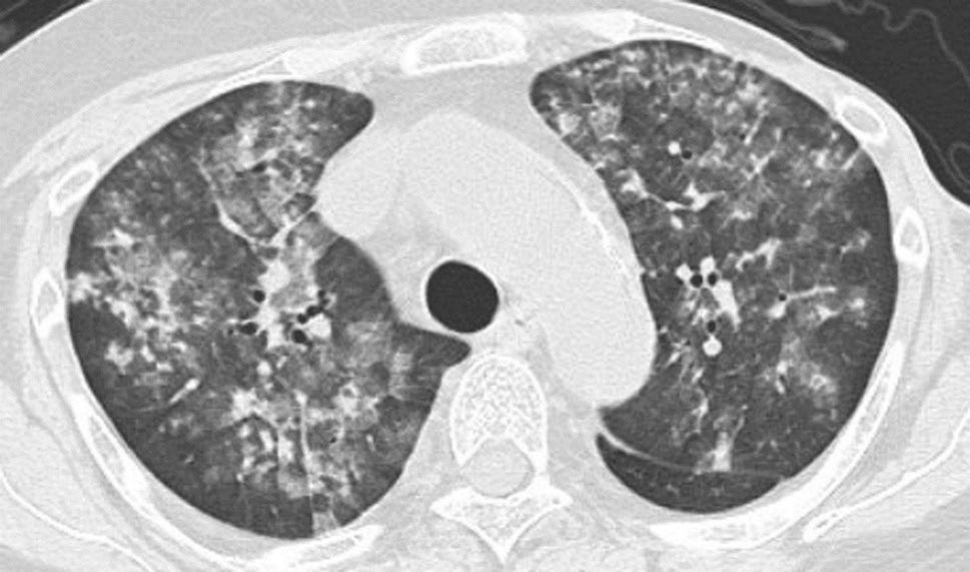
A 56-year-old woman with diffuse alveolar hemorrhage under treatment for thrombocytopenia and uterine cancer. High-resolution computed tomography shows extensive ground-glass attenuation with multiple nodular lesions

Table 1 shows the characteristics of patients and the diagnostic methods. Age, underlying disease or condition, and diagnostic methods were significantly different among the groups (*p* < 0.05). Table 2 shows the analysis of HRCT findings of pulmonary complications using the *χ*^*2*^ test. The interobserver agreement was 0.374–0.775 (fair-good). There were significant differences in consolidation (*p* = 0.041), nodule (*p* < 0.001), BVB thickening (*p* < 0.001), ILS thickening (*p* < 0.001), and axial distribution (*p* = 0.044) among the groups. For PCP, the frequencies of consolidation (37.8%), nodule (14.6%), BVB thickening (8.5%), ILS thickening (48.8%), and outer distribution (2.4%) were significantly low, and the frequency of diffuse axial distribution (87.8%) was significantly high among the groups (Fig. 1). For drug-induced pneumonia, the frequencies of ILS thickening (89.5%) and outer distribution (26.3%) were significantly high (Fig. 2) and the frequency of diffuse axial distribution (63.2%) was significantly low among the groups. The ILS thickening was also frequent in other noninfectious disease; IPS, DAH, and pulmonary edema (100%, respectively) and in infectious disease; CMV-P and fungal infection (66.7%, respectively) (Table 2). The frequency of BVB thickening was significantly high among patients with bacterial pneumonia (66.7%) (Fig. 3) and those with pulmonary edema (100%). The frequency of nodules was significantly high among patients with DAH (75.0%) (Fig. 4), fungal infection (100%), and TB (100%).

**Table 2:** The HRCT findings

		PCP (*n* = 82)	Drug induced pneumonia (*n* = 38)	Bacterial pneumonia (*n* = 9)	CMV-P (*n* = 6)	IPS (*n* = 6)	DAH (*n* = 4)	Fungal infection (*n* = 3)	TB (*n* = 2)	Pulmonary edema (*n* = 2)	*P* value	*k* value
GGA-crazy-paving		41 (50.0%)	20 (52.6%)	5 (55.6%)	4 (66.7%)	4 (66.7%)	3 (75.0%)	2 (66.7%)	0 (0%)	0 (0%)	0.574	0.437
GGA-mosaic		63 (76.8%)	22 (57.9%)	4 (44.4%)	4 (66.7%)	4 (66.7%)	3 (75.0%)	2 (66.7%)	1 (50.0%)	1 (50.0%	0.458	0.462
Consolidation		31 (37.8%)**	19 (50.0%)	7 (77.8%)	5 (83.3%)	4 (66.7%)	3 (75.0%)	2 (66.7%)	0 (0%)	2 (100%)	0.041	0.595
Nodules		12 (14.6%)**	9 (23.7%)	3 (33.3%)	3 (50.0%)	2 (33.3%)	3 (75.0%)*	3 (100%)*	2 (100%)*	0 (0%)	< 0.001	0.487
Nodule size	Micro Small Large	2 (2.4%) 5 (6.1%) 5 (6.1%)	5 (13.2%) 3 (7.9%) 1 (2.6%)	1 (11.1%) 1 (11.1%) 1 (11.1%)	0 (0%) 3 (50.0%) 0 (0%)	1 (16.7%) 1 (16.7%) 0 (0%)	3 (75.0%) 0 (0%) 0 (0%)	2 (66.7%) 0 (0%) 1 (33.3%)	2 (100%) 0 (0%) 0 (0%)	–	0.140	0.444
Nodule distribution	Centrilobular Perilymphatic Random	4 (4.9%) 0 (0%) 8 (9.8%)	6 (15.8%) 1 (2.6%) 2 (5.3%)	2 (22.2%) 0 (0%) 1 (11.1%)	2 (33.3%) 0 (0%) 1 (16.7%)	0 (0%) 0 (0%) 2 (33.3%)	3 (75.0%) 0 (0%) 0 (0%)	2 (66.7%) 0 (0%) 1 (33.3%)	1 (50.0%) 0 (0%) 1 (50.0%)	–	0.582	0.478
BVB thickening		7 (8.5%)**	12 (31.6%)	6 (66.7%)*	1 (16.7%)	3 (50.0%)	2 (50.0%)	2 (66.7%)	0 (0%)	2 (100%)*	< 0.001	0.485
ILS thickening		40 (48.8%)**	34 (89.5%)*	3 (33.3%)	4 (66.7%)	6 (100%)	4 (100%)	2 (66.7%)	0 (0%)	2 (100%)	< 0.001	0.539
Axial distribution	Inner Outer Diffuse Indeterminate	4 (4.9%) 2 (2.4%)** 72 (87.8%)* 4 (4.9%)	2 (5.3%) 10 (26.3%)* 24 (63.2%)** 2 (5.3%)	0 (0%) 0 (0%) 8 (88.9%) 1 (11.1%)	1 (16.7%) 1 (16.7%) 4 (66.7%) 0 (0%)	1 (16.7%) 2 (33.3%) 3 (50.0%) 0 (0%)	1 (25.0%) 0 (0%) 3 (75.0%) 0 (0%)	0 (0%) 0 (0%) 3 (100%) 0 (0%)	0 (0%) 0 (0%) 2 (100%) 0 (0%)	1 (50.0%) 0 (0%) 1 (50.0%) 0 (0%)	0.044	0.374
Craniocaudal distribution	Upper Lower Diffuse Indeterminate	3 (3.7%) 3 (3.7%) 72 (87.8%) 4 (4.9%)	2 (18.2%) 4 (36.4%) 2 (18.2%) 3 (27.3%)	1 (11.1%) 0 (0%) 7 (77.8%) 1 (11.1%)	0 (0%) 0 (0%) 6 (100%) 0 (0%)	0 (0%) 2 (33.3%) 4 (66.7%) 0 (0%)	0 (0%) 0 (0%) 4 (100%) 0 (0%)	0 (0%) 0 (0%) 3 (100%) 0 (0%)	0 (0%) 0 (0%) 2 (100%) 0 (0%)	0 (0%) 0 (0%) 2 (100%) 0 (0%)	0.819	0.403
LN swelling		21 (25.6%)	9 (23.7%)	1 (11.1%)	3 (50.0%)	1 (16.7%)	2 (50.0%)	0 (0%)	1 (50.0%)	1 (50.0%)	0.568	0.692
Effusion		26 (31.7%)	15 (39.5%)	6 (66.7%)	2 (33.3%)	4 (66.7%)	2 (50.0%)	2 (66.7%)	0 (0%)	2 (50.0%)	0.233	0.775

*PCP* pneumocystis pneumonia, *TB* tuberculosis, *CMV-P* cytomegalovirus pneumonia, *IPS* idiopathic pneumonia syndrome, *DAH* diffuse alveolar hemorrhage, *GGA* ground-glass attenuation, *BVB* bronchovascular bundle, *ILS* interlobular septum, *LN* lymph node

*Significantly higher (adjusted standard residuals > 1.96) in groups, **Significantly lower (adjusted standard residuals < – 1.96) in groups

The multiple logistic regression analyses identified significant indicators for each disease (Table 3). The absence of nodules (*p* = 0.015; odds ratio [OR], 2.990; 95% confidence interval [CI], 1.239–7.214; sensitivity, 85.4%; specificity, 35.7%), the absence of BVB thickening (*p* = 0.001; OR, 5.158; 95% CI, 1.983–13.414; sensitivity, 91.5%; specificity, 40.0%), and the absence of ILS thickening (*p* = 0.002; OR, 3.322; 95% CI, 1.527–7.228; sensitivity, 51.2%; specificity, 78.6%) were identified as indicators of PCP (Tables 3 and 4). The presence of ILS thickening (*p* < 0.001; OR, 7.385; 95% CI, 2.460–22.174; sensitivity, 89.5%; specificity, 46.5%) was identified as an indicator of drug-induced pneumonia (Tables 3 and 4). The presence of BVB thickening (*p* = 0.005; OR, 10.059; 95% CI, 1.013–99.863; sensitivity, 66.7%; specificity, 79.7%) was identified as an indicator of bacterial pneumonia (Tables 3 and 4). The presence of nodules (*p* = 0.049; OR, 10.059; 95% CI, 1.013–99.863; sensitivity, 75.0%; specificity, 77.0%) was identified as an indicator of DAH (Tables 3 and 4). The AUC values were 0.605–0.760.

**Table 3:** The results of the multiple logistic regression analysis

HRCT findings		PCP (*n* = 82)	Non-PCP (*n* = 70)	Wald	Odds ratio [95% CI]	*p* value
Nodule	Positive Negative	12 (14.6%) 70 (85.4%)	25 (35.7%) 45 (64.3%)	5.940	– 2.990 [1.239, 7.214]	0.015
BVB thickening	Positive Negative	7 (8.5%) 75 (91.5%)	28 (40.0%) 42 (60.0%)	11.316	– 5.158 [1.983, 13.414]	0.001
ILS thickening	Positive Negative	40 (48.8%) 42 (51.2%)	55 (78.6%) 15 (21.4%)	9.162	– 3.322 [1.527, 7.228]	0.002

		Drug induced pneumonia (*n* = 38)	Non-drug induced pneumonia (*n* = 114)	Wald	Odds ratio [95% CI]	*p* value
ILS thickening	Positive Negative	34 (89.5%) 4 (10.5%)	61 (48.3%) 53 (46.5%)	12.705	7.385 [2.460, 22.174] –	< 0.001

		Bacterial pneumonia (*n* = 9)	Non-bacterial pneumonia (*n* = 143)	Wald value	Odds ratio [95% CI]	*p* value
BVB thickening	Positive Negative	6 (66.7%) 3 (33.3%)	29 (20.3%) 114 (79.7%)	7.827	7.862 [1.854, 33.337] –	0.005

		DAH (*n* = 4)	Non-DAH (*n* = 148)	Wald	Odds ratio [95% CI]	*p* value
Nodule	Positive Negative	3 (75.0%) 1 (25.0%)	34 (23.0%) 114 (77.0%)	3.885	10.059 [1.013, 99.863] –	0.049

*PCP* pneumocystis pneumonia, *BVB* bronchovascular bundle, *ILS* interlobular septum, *DAH* diffuse alveolar hemorrhage, *CI* confidence interval

**Table 4:** Sensitivity, specificity, accuracy, PPV, and NPV of each HRCT finding for detecting each infection

	Sensitivity	Specificity	PPV	NPV	AUC
PCP					
Absence of nodules	85.4%	35.7%	60.9%	67.6%	0.605
Absence of BVB thickening	91.5%	40.0%	64.1%	80.0%	0.657
Absence of ILS thickening	51.2%	78.6%	73.7%	57.9%	0.649
Drug induced pneumonia					
Presence of ILS thickening	89.5%	46.5%	35.8%	93.0%	0.680
Bacterial pneumonia					
Presence of BVB thickening	66.7%	79.7%	17.1%	97.4%	0.732
DAH					
Presence of nodules	75.0%	77.0%	8.1%	99.1%	0.760

*PPV* positive predictive value, *NPV* negative predictive value, *PCP* pneumocystis pneumonia, *BVB* bronchovascular bundle, *ILS* interlobular septum, *DAH* diffuse alveolar hemorrhage, *AUC* area under the curve

## Discussion

Our study suggested that differentiation between pulmonary complications with acute or subacute extensive GGA in immunocompromised patients might be possible by evaluating various HRCT findings. Extensive GGA often causes difficulty in making a clinical diagnosis, especially in immunocompromised patients; however, our results may be useful for an early and correct diagnosis and for selecting appropriate treatment.

In patients with PCP, the frequencies of consolidation, nodules, BVB thickening, ILS thickening, and outer distribution were significantly low and the frequency of diffuse axial distribution was significantly high in comparison to the other groups in our study. In particular, the absence of nodules and the absence of BVB thickening could be indicators for differentiation. In PCP, nodules have been reported as infrequent findings [9, 10], which may support the results of the present study.

In patients with drug-induced pneumonia, the frequencies of ILS thickening and outer distribution were significantly high in comparison to the other groups in our study. ILS thickening is seen in lymphatic or infiltrative disease. It is frequently observed in drug-induced pneumonia [11]; however, it is also a common finding in pulmonary edema and was observed in 2 patients with pulmonary edema in this study (100%). In addition, the frequency of ILS thickening was high in other noninfectious diseases including IPS and DAH and infectious diseases including CMV-P and fungal infection in this study (66.7–100%), with a small number of subjects. Thus, it may be a non-specific finding among pulmonary complications with extensive GGA.

Bilateral diffuse opacities can be observed in immunocompromised patients with bacterial pneumonia [12]. In patients with bacterial pneumonia, the frequency of BVB thickening was significantly high and BVB thickening could be used as an indicator in our study. The BVB is the connective tissue sheath that encloses the bronchi and hilar vessels; thus, BVB thickening includes thickening of the bronchial wall and bronchovascular interstitium [13]. Several studies reported that bronchial wall thickening due to inflammatory reactions was frequently observed in patients with bacterial pneumonia [14–16]. The frequency was also relatively higher in patients with a fungal infection, IPS, and DAH; thus, it could be a nonspecific finding among the pulmonary complications with extensive GGA.

Among the groups in our study, nodules were significantly frequent in the DAH, fungal infection, and TB groups. It may be an indicator in DAH. Diffuse nodular opacities and patchy GGA have been reported to be the most common findings in DAH and nodules are observed due to deposition of hemosiderin-laden macrophages in alveolar spaces and small vessels, or capillaritis [17, 18]. In TB, nodules are a frequent CT finding in both immunocompetent and immunocompromised patients [19]. In fungal infections, including invasive aspergillosis and infections other than aspergillosis, the predominant CT findings include nodules [20–23]. These reports support our study. However, according to our results, when small nodules are observed coexisting with extensive GGA, the possibility of DAH should be considered.

There were no significant differences in CMV-P or IPS among the groups in our study. HRCT findings of CMV-P have been described as being a mixture of patterns with GGA, consolidation, and small nodules [24, 25]. Our study also supported the findings of the previous studies and these various findings could make it difficult to diagnose CMV-P in patients with extensive GGA. CMV-P and PCP are often difficult to distinguish because of the clinical and radiological similarities. HRCT findings for the differential diagnosis of CMV-P and PCP included extensive GGA with a mosaic pattern and an apical distribution in PCP, and an ill-defined demarcation of GGA, consolidation, and nodules in CMV-P [26, 27]. IPS included a variety of clinical, histological, and radiographic patterns [4, 5]. This could cause difficulty in the diagnosis of IPS among patients with extensive GGA.

Our study was associated with several limitations. First, the present study was retrospective in nature; thus, the CT protocols and diagnostic procedures were diverse. Second, the number of cases of diseases other than PCP and drug-induced pneumonia was relatively small. The number of HIV-infected patients was also small due to the single-center nature of the study. This may have affected the CT findings especially in PCP patients. The patients with viral pneumonia other than CMV-P were not included in this study. The recent SARS-COV-2 infection was not evaluated in this study though it is frequent in the present and could continue in the future. Third, the reliability of the diagnosis may be controversial because pathological confirmation and the resultant radiologic-pathologic correlation was only available for a small number of cases. However, it is often difficult for immunocompromised patients to undergo invasive procedures. More than one disease can coexist in extensive GGA in clinical practice; however, our study population was limited to patients with only one final diagnosis. Therefore, there is no guarantee that the results of this study always apply to clinical cases.

In conclusion, differentiation between pulmonary complications with extensive GGA in immunocompromised patients may be possible using HRCT findings. Nodules, BVB thickening, and ILS thickening were found to be particularly useful HRCT findings.
